# Outdoor advertising, obesity, and soda consumption: a cross-sectional study

**DOI:** 10.1186/1471-2458-13-20

**Published:** 2013-01-10

**Authors:** Lenard I Lesser, Frederick J Zimmerman, Deborah A Cohen

**Affiliations:** 1 Department of Health Policy, Palo Alto Medical Foundation Research Institute, Palo Alto, CA, USA; 2 Department of Health Services, School of Public Health, University of California, Los Angeles, USA; 3 RAND Corporation, Santa Monica, CA, USA

**Keywords:** Obesity, Sugar-sweetened beverages, Advertising

## Abstract

**Background:**

Recent research has shown that neighborhood characteristics are associated with obesity prevalence. While food advertising in periodicals and television has been linked to overweight and obesity, it is unknown whether outdoor advertising is related to obesity.

**Methods:**

To test the association between outdoor food advertising and obesity, we analyzed telephone survey data on adults, aged 18–98, collected from 220 census tracts in Los Angeles and Louisiana. We linked self-reported information on BMI and soda consumption with a database of directly observed outdoor advertisements.

**Results:**

The higher the percentage of outdoor advertisements promoting food or non-alcoholic beverages within a census tract, the greater the odds of obesity among its residents, controlling for age, race and educational status. For every 10% increase in food advertising, there was a 1.05 (95% CI 1.003 - 1.093, p<0.03) greater odds of being overweight or obese, controlling for other factors. Given these predictions, compared to an individual living in an area with no food ads, those living in areas in which 30% of ads were for food would have a 2.6% increase in the probability of being obese.

**Conclusions:**

There is a relationship between the percentage of outdoor food advertising and overweight/obesity.

## Background

Obesity is one of the world’s most intractable health problems [1]. While the causes of obesity are multifactorial, a growing body of evidence implicates food marketing as a major contributor to the epidemic [2,3]. Recent comprehensive reviews leave no doubt that a variety of marketing strategies increases food consumption in the laboratory environment and in natural settings [4-6]. Yet the public health community does not know how large an effect food marketing has on population-level obesity status. Additionally, there is limited research on advertising’s effect on consumption and obesity in adults outside the laboratory setting.

There is ample evidence that, in the words of the Institute of Medicine, “advertising works” [7]. Marketing research demonstrates that as marketing expenditures to promote a particular food increase, so do purchases of those foods [8-10]. Industry marketing campaigns can even counter the effect of health promotion messages. For instance, in response to the growing public health concern over cholesterol and a decrease in butter sales, a marketing campaign to promote butter in Canada had a positive effect on butter demand [11].

Many researchers have focused on the content of food television advertising and its association with consumption and obesity [12-16]. The public health community has focused little attention to the role of outdoor advertising on food consumption and obesity. One important driver of differences in obesity rates among socioeconomic and racial and ethnic groups [17] may be the variation in their exposure to outdoor food advertising. A recent study directly observed outdoor advertisements related to food and physical activity in several diverse zip codes in New York, Philadelphia, Los Angeles, and Austin [18]. The density of advertising in zip codes whose residents were predominantly African American was highest; Latino zip code areas had slightly lower densities; zip code areas with predominantly white residents had the lowest densities of all. This study further found that living in a high SES zip code, regardless of its residents' predominant race or ethnicity, was generally protective against exposure to obesity-promoting outdoor advertising (food, fast food, sugary beverages, sedentary entertainment, and motorized transportation).

Given that food marketing predominantly promotes foods that discouraged by the Dietary Guidelines for Americans [19] differences in exposure to outdoor food advertising by SES and race/ethnicity may partly explain observed racial/ethnic disparities in obesity rates. This study investigated whether individuals living in areas with higher proportions of outdoor food advertising, compared to those in living in areas with lower amounts, have greater odds of obesity and a higher rate of soda consumption.

## Methods

### Data sources

We analyzed outdoor advertisement data collected from a study on alcohol consumption that was geographically limited to allow site visits [20]. The areas selected were densely populated (>2000 residents per square mile) tracts within a one-hour drive from Drew Medical Center in Los Angeles and within 2 hours from Tulane University in New Orleans. From those areas (1328 tracts in Los Angeles; 381 tracts in Louisiana), a random sample of 114 census tracts in Los Angeles County and 114 census tracts in Southeastern Louisiana were selected. Observers in two-person teams visited sampled census tracts from September 2004 to August 2005 in Southern Louisiana and from October 2004 to November 2005 in Los Angeles County. They systematically surveyed each tract once, first following the perimeter of each tract and then going through each street in the tract from north to south and from east to west. Observers recorded each outdoor advertisement’s latitude and longitude using a GPS monitor.

Each team then followed a standard protocol and used a standard data collection instrument to document the type of the product advertised. This information was used to classify all the outdoor advertisements into 4 major product subcategories: alcohol, tobacco, food and/or restaurants, and other products. Observers also recorded the format of media used: 1) posters, flyers, flags, banners, or transit shelters or benches, 2) small billboards (larger than poster or banners but smaller than 12’ × 24’), 3) average size billboards (12’ × 24’), and 4) extra-large billboards (14’ × 48’). The surveyors included all types of outdoor advertisements, except for storefront advertisements, as they were part of a separate study. In order to better capture double-sided billboards and multiple banners, posters, and flyers at the same advertising location, observers coded the frequency with which the ad appeared: Once, 2–4 times, 5–10 times, 11+ times. A quality control supervisor conducted separate concurrent observations in approximately 10% of the selected census tracts in both sites to ensure the reliability of observations. The supervisor’s results were compared to the field staff’s to verify agreement, which was routinely greater than 0.8.

During the same time period and in the same census tracts in which the outdoor advertisements were surveyed, telephone interviews were conducted with a systematic sample of adults from geographically referenced telephone-listed households. Calling was halted early in New Orleans due to Hurricane Katrina, when 106 tracts had been completed. Participants were offered $15 to complete a 15–20 minute interview. The interview asked many health questions, and included the participants’ height, weight, and how many 12-oz sodas they consumed in the past 24 hours.

In Louisiana the average response rate per census tract was 37.9%; in Los Angeles, it was 34.4%. For comparison, the response rate for California and Louisiana in the 2005 wave of the Behavioral Risk Factor Surveillance Survey was 29.2% and 36.5%, respectively [21]. There were 2,881 respondents in our study. We excluded adults who were underweight (BMI<18.5, n=48), because we were concerned that this could have reflected an illness, rendering them less susceptible to obesity. Our main analysis and demographic data contain information on the 2589 participants without missing data.

### Statistical methods

Our main outcome was self-reported BMI categorized according to National Institutes of Health criteria (normal weight, overweight, or obese) [22]. A secondary outcome variable was the number of 12-oz sodas consumed in the previous 24 hours.

Advertisements were coded into one of several mutually exclusive categories, including entertainment, food/beverage (not including alcohol), alcohol, and other products. We calculated the percentage of the advertisements in each census tract that promoted all types of food or non-alcoholic beverages. We did not distinguish between healthy and unhealthy food advertisements, as fewer than 5% of the ads were for vegetables or fruits. Most were for drinks, snacks, and restaurants. Additionally, both healthy and unhealthy food advertisements are likely to cue the body to eat and stimulate hunger [23].

As a measure of effective exposure to food ads, we used the percentage of total advertising that was for food. Other authors have used this measure when analyzing outdoor advertising [18]. This percentage-based measure is useful because the effect of advertising is reduced when it must compete with other advertising. That is, a tract with 50 food ads out of 100 ads total will have an effective exposure to food ads that is much higher than a tract that has 50 food ads out of 1,000 total ads. Accordingly the percentage reflects the strength of food advertising exposure, while also controlling for zoning laws that may limit the total number of all types of advertisements in an area. Additionally, the percentage-based measure was used instead of looking at percentage of sheet space because the former adjusts for physical proximity to the ad and for the speed with which consumers view ads. For instance, billboards have more sheet space, but are often viewed while driving, and thus are viewed for a shorter period of time. Bus stop ads have less sheet space; individuals view them for a longer period of time, while waiting for a bus or driving by them at slower speeds.

We coded those census tracts with no advertisements at all as having 0% of ads related to food. To enhance interpretability of the scale of the association, we divided the food-ad percentage measure by 10, so that a 1-unit increase in the main independent variable represents a 10-percentage-point increase in the percent of ads devoted to food.

We included several covariates in our model including age in years and years-squared (to increase the fit of our model), education, race, and ethnicity as potential confounders. Education was dummy coded, with college education as the baseline. We included race and ethnicity in the model as exclusive categories. For respondents that indicated more than one race category (approximately 2%), we used the first one recorded by the surveyor. To control for the total number of advertisements in each census tract, we included the total count of all advertisements for each census tract in the model. We accounted for the respondents’ clustering in census tracts by using STATA’s *svy* command.

We also examined which census-tract level factors were associated with the percentage of food advertisements. We used 2000 census data and defined census tracts based on predominant race/ethnicity and income. Each census tract was labeled with the race that corresponded to the most prevalent race or ethnicity in that census tract. We further categorized the census tracts as low or high income by whether their median incomes were below or above the median income for the sampled census tracts in their region (i.e. Louisiana or Los Angeles). We categorized the census tracts instead of trying to model a linear relationship because the former is recommended on efficiency grounds when the underlying relationship is non-linear. We used logistic regression to determine which factors were associated with a census tract had any food advertisements. We conducted an ordered logistic regression to test the hypothesized association between overweight/obesity and advertising in a fully adjusted model.

To facilitate interpretation, we simulated the probabilities of obesity for adults living in areas with different amounts of food advertising. Doing so permits the comparison of estimated obesity rates in a hypothetical census tract with no food advertising to one with high levels. To perform this simulation, we used our regression model and held all covariates at their mean level. Then, using the regression equation, we predicted the probability of obesity at food advertising levels of 0% and 30%. The values of 0% (no food advertising) and 30% correspond to the 10^th^ and 90^th^ percentile values of percentage of all advertisements promoting food in the sampled census tracts. These values also correspond to two representative regions in the regions we studied: West Los Angeles or Old Aurora in New Orleans (which have generally higher median incomes and few minorities) and South Los Angeles or an urban area of the Seventh Ward in New Orleans (which have lower median incomes and more minorities).

Because previous work has shown that advertisements for sugary drinks were one of the most prevalent forms of outdoor advertising [18], we hypothesized that the amount of general advertising would likely be associated soda drinking. We used count data models to assess the association of the number of soda drinks per day with food advertising. As above, we conducted simulations to compare predicted soda consumption in two hypothetical census tracts.

All analyses were performed using STATA, version 11. The RAND Human Subjects Protection Committee approved the study.

## Results

Table 1 shows the demographic characteristics of the sample used in our ordered BMI analysis. Twenty-five percent (25%) of the adults were obese, while 35% were overweight and 40% were of normal weight. Adults drank, on average, 1.3 (sd 1.9) 12-ounce sodas per day.

**Table 1:** Demographic information on sample used in main analysis (n=2589)

	**Number**	**Percent of Sample or Mean (SD)**
Age		43.5 (13.5)
Weight Status		
Normal Weight	1028	40%
Overweight	909	35%
Obese	652	25%
Gender		
Male	975	38%
Female	1614	62%
Highest Grade Completed		
Less Than High School	88	3%
Less than 12th Grade	210	8%
High School Graduate	630	24%
Some College Education	647	25%
College Graduate	1010	39%
Ethnicity		
Not Hispanic	1796	81%
Hispanic	497	19%
Race		
White	1306	50%
Black	793	31%
Asian	92	4%
Other Race	398	15%
Servings of soda in the last day		1.3 (1.9)

We included 219 census tracts in the analysis. (One tract was deleted due to its being an industrial area, with only one respondent). The average number of outdoor advertisements in each census tract was 10.2 (sd=17.3, median=4). The average percentage of the advertisements related to food or beverages was 10.4% (sd=17.5, median=0). Among the census tracts, 67 (30.6%) had no outdoor advertisements, and 122 (55.7%) had no food advertisements. Los Angeles had significantly more ads per census tract than New Orleans (14 vs. 6, p<0.01), but had a lower percentage of food ads than New Orleans (6% vs. 15%, p<0.05).

The median household income in the sampled tracts in Louisiana was $34,930; in Los Angeles it was $41,957. Census tracts that were low-income with the plurality of their population designated as Black or Latino had significantly greater odds of having *any* food ads compared to high-income white census tracts. (Table 2) We found no significant relationship between census tract characteristics and *percent* of outdoor advertisements promoting food.

**Table 2:** **Predictors of having any food advertisements in a census tract**^**a**^

	**Odds Ratio**	**95% CI**
Socio-Economic Characteristics		
High Income White	1.00	-
Low Income White	1.52	0.52-4.41
High Income Black	2.94	0.83-10.35
Low Income Black	2.59	1.04-6.48
High Income Latino	0.93	0.21-4.21
Low Income Latino	3.10	1.03-9.20
High Income Asian	6.34	0.61-66.2
Low Income Asian	2.15	0.38-12.19

^a^Regression also controls for percent female, age and age-squared (not shown).

The ordered logistic regression revealed that those who lived in areas with a greater percentage of food advertisements had increased odds of overweight and obesity. (Table 3) For every 10% increase in food advertisements, the odds of being obese increased by 5% [OR 1.05 (95% CI: 1.00, 1.10), p<0.03]. Advertising clutter (the total number of advertisements in the census tract) did not significantly confound this relationship.

**Table 3:** **Odds of obesity in relation to percentage of food advertisements**^**a**^

	**Odds Ratio**	**95% CI**
Food Ad Percentage (in 10% increments)	1.05	1.00-1.10^b^
Total Number of Ads	1.00	1.00-1.00
Demographic Control Variables		
Female Gender	0.65	0.56-0.76
Education		
Less Than High School	1.99	1.29-3.07
Some High School	1.54	1.16-2.02
High School	1.38	1.14-1.67
Some College	1.33	1.09-1.61
College	1.00	-
Ethnicity		
Not Hispanic	1.00	-
Hispanic	1.38	1.08-1.76
Race		
White	1.00	-
Black	2.22	1.83-2.71
Asian	0.57	0.36-0.89
Other Race	1.31	0.99-1.76

^a^Model also controls for age and age-squared (not shown). ^b^p<0.03: CI overlaps 1.00 due to rounding.

Using a simulation, and controlling for individual factors such as race/ethnicity and education, we predicted that in an area with no food ads, 37.1% of adults would be overweight and 22.6% would be obese. In an area with 30% food ads, 38.0% would be overweight and 25.2% would be obese. Given these predictions, compared to an individual living in an area with no food ads (e.g. West Los Angeles or Old Aurora), those living in areas with 30% food ads (e.g., South Los Angeles or an urban area of the Seventh Ward) would have a 2.6% increase in the probability of being obese. In a census tract with 5000 people, if 30% of ads promote food, we would expect to find an additional 100–150 people who are obese.

We then tested the association between soda drinking and food advertisements, using a count model. We performed several tests before picking the appropriate count model. The likelihood test for the assumption of equidispersion was significant, rejecting the poisson model and suggesting a negative binomial model is preferred. The Vuong test was not significant, failing to reject the assumption of no excess zeros, suggesting that a negative binomial model is preferred to a zero-inflated negative binomial model. Thus, our final model used the negative binomial model.

The percent of food ads was also associated with the number of 12-ounce sodas the respondents drank in the last day (Table 4). Each 10% increase in food advertisements was associated with a 6% increase in the number of sodas consumed [IRR=1.06 (95% CI: 1.03-1.09, p<0.01)]. A simulation showed that compared to a tract with 0% food advertising, people in a tract with 30% advertising would be drinking 0.2 more sodas per day. This translates to about 16.8 fluid ounces, or about 196 kilocalories per week.

**Table 4:** **Rate of soda consumption in relation to food advertisements**^**a**^

	**Incidence Rate Ratio**	**95% CI**
Food Ad Percentage (in 10% increments)	1.06	1.03-1.09
Total Number of Ads	1.00	0.99-1.03
Demographic Control Variables		
Female Gender	0.76	0.68-0.85
Education		
Less Than High School	1.18	0.89-1.56
Some High School	1.61	1.29-2.01
High School	1.32	1.15-1.51
Some College	1.09	0.95-1.25
College	1.00	-
Ethnicity		
Not Hispanic	1.00	-
Hispanic	0.71	0.59-0.85
Race		
White	1.00	-
Black	0.97	0.85-1.11
Asian	0.42	0.30-0.58
Other Race	0.85	0.72-1.01

^a^Model also controls for age, age-squared (not shown).

## Discussion

Advertising can influence food consumption by directly promoting food purchasing and indirectly by influencing social norms. Food advertising is associated with obesity in children [24-27] and marketing more generally has been identified as one of the major factors contributing to the obesity epidemic [2].

Our research confirms the observations of previous studies, which documented that unhealthy food advertisements are more prevalent in low income and minority communities [18,28]. We also found different amounts of advertising in the two cities we surveyed. This higher number of ads, but lower percentage of food ads in Los Angeles, likely reflects the city’s focus on entertainment, as documented in another study [18].

Most research on the effect of outdoor advertising has focused on tobacco and alcohol. For instance, many studies have shown that African-American neighborhoods (regardless of community income) have more alcohol and tobacco outdoor ads than White areas [29,30]. In a study similar to ours, researchers found that exposure to outdoor alcohol advertising was associated with problem drinking in women [31].

Most of the other evidence around the effect of advertising has looked at children, who are likely more susceptible to advertising [7]. Alcohol advertisements have been found to cluster around schools and predicted alcohol consumption intentions by children at those schools [32]. Children exposed to tobacco advertising are more likely to smoke [33]. Other research has shown that unhealthy ads (including food, tobacco, and alcohol) have clustered around child-serving institutions [28]. In the area of obesity, many studies have documented an association between various types of advertising and consumption of unhealthy foods and obesity [12,24,34,35]. One other study looked at restaurant and food store signage and its relationship to obesity in adults living in the surrounding area [36]. The authors found that while advertisements for unhealthy foods around restaurants were associated with obesity, signage around convenience stores was not associated with obesity.

Thus, the summary of research in other areas points to an effect of outdoor advertising on the intentions of the viewers of those ads. This analysis finds parallel results to the previous research on alcohol, tobacco, and food: those who live in areas with higher percentages of food advertising have greater odds of obesity than those living in areas with a lower percentage of food ads. While we controlled for several factors that were likely to confound the association, there may be other unmeasured variables (e.g. individual preferences, urbanization of census tract, roadway structure, exposure to television advertisements) that could explain the association.

Our study has limitations. We used self-reported information on height, weight, and soda drinking. Self-reports of dietary practices are always subject to recall bias, but 24-hour recalls seem to be better than longer-term food frequency questionnaires [37]. BMI data are likely under-reported by about 1 unit (kg/m^2^) [38], which could have shifted the BMI distribution in our study downward. If respondents in our study reported a lower BMI than was true, we would be less likely to find an association between obesity and advertisements. Thus, the actual association may be stronger than the one we report.

Our data is also limited to two metropolitan areas. Further studies should try to replicate these results in different geographic settings. The survey data were collected by phone surveys. While the response rates were similar to those in another large national survey, our results could be biased by lack of response from those without landlines. However, one study found that the odds of obesity were similar in those with landlines and wireless phones [39]. While those without any phone service may have a higher odds of obesity than those with a landline, the proportion of the population in this category is less than 2% [39]. If those without phone service have a higher prevalence of obesity and are likely to live in areas where minorities predominate, and thus advertisements are prevalent, our effect estimates are likely to be biased towards the null hypothesis.

Furthermore, research on outdoor advertising is limited by the difficulty in determining who is actually exposed to the advertisements over a particular time period. We assumed that people were exposed to advertisements in their own neighborhoods. However, people may also be exposed to advertisements on their way to work and other destinations; advertisements and exposures may also change over time. Our findings indicate that the exposure to food ads around a person’s home does measure some aspect of the food environment that is associated with obesity. Future studies taking into account work location, time spent at work/home, and means of transportation to work may find a stronger association. Additionally, future studies could use actual locations of respondents to evaluate whether distance to advertisements matters.

Our independent variable, percent of food advertisements devoted to food, has some limitations. In census tracts where the total number of ads is small, but all (100%) of the ads are for food, we may have overestimated the impact of food ads. However, we controlled for the total number of ads in a tract, which may also be a measure of census tract geographic size. Even if there are only two ads in a geographically small census tract, they could be highly impactful in promoting consumption.

When looking at associations between individuals and their neighborhood’s characteristics, there is always a possibility of self-selection bias. People may deliberately move into neighborhoods in which particular foods are advertised. However, this is unlikely to be the dominant reason for selection of a neighborhood. Additionally, evidence from research on physical activity and the built environment suggests that self-selection has a weak influence on associations with the built environment [40].

## Conclusions

An important contribution of this work is to demonstrate that outdoor advertising is associated with a modest, but clinically meaningful, increased likelihood of obesity. The reasonable way to prove a causal relation would be to reduce outdoor food advertising in certain neighborhoods and determine whether obesity rates change. Given the health crisis associated with obesity, such measures may be warranted.

If the above associations are confirmed by additional research, policy approaches may be important to reduce the amount of food advertising in urban areas. Bans on certain kinds of alcohol ads have reduced consumption in many countries [41]. Although efforts to control the placement of a particular type of outdoor advertising are likely to be deemed unconstitutional in the United States, requiring warnings on those advertisements are likely to be constitutionally acceptable [42]. Innovative strategies, such as warning labels, counter-advertising, or a tax on obesigenic advertising should be tested as possible public health interventions for reducing the prevalence of obesity.

## Competing interest

The authors declare that they have no competing interests.

## Authors’ contributions

LL led the study, performed the analysis, and wrote the manuscript. FZ provided statistical input and did extensive editing of the manuscript. DC provided the dataset and did extensive editing of the manuscript. All authors read and approved the final manuscript.

## Pre-publication history

The pre-publication history for this paper can be accessed here:

http://www.biomedcentral.com/1471-2458/13/20/prepub
